# Waist Circumference as Measure of Abdominal Fat Compartments

**DOI:** 10.1155/2013/454285

**Published:** 2013-05-08

**Authors:** Scott M. Grundy, Ian J. Neeland, Aslan T. Turer, Gloria Lena Vega

**Affiliations:** ^1^Departments of Internal Medicine, Clinical Nutrition and Center for Human Nutrition, University of Texas Southwestern Medical Center, Dallas, TX, USA; ^2^Division of Cardiology, University of Texas Southwestern Medical Center, Dallas, TX, USA

## Abstract

This study examines intercorrelations among waist circumference (WC), intraperitoneal fat (IPF), and subcutaneous abdominal fat (SAF) in ethnically diverse Dallas Heart Study consisting of 1538 women and 1212 men (50% Black). Correlations between fat depots and triglyceride or HOMA2-IR, biomarkers of metabolic syndrome, are also reported. Total abdominal fat (TAF), ASF, and IPF masses were measured by magnetic resonance imaging. The highest correlations with WC according to ethnicity and gender were noted for TAF (*R* ^2^ = 0.81 − 0.88) with progressively lower correlations with ASF (0.65–0.82) and IPF (0.29–0.85). The percentage of IPF relative to TAF was not significantly correlated with WC. For all WC categories, higher IPF/ASF ratios were associated with higher triglyceride levels. In contrast, differences in ratios had little or no association with HOMA2-IR. However, when all data were pooled, IPF was positively correlated with both triglyceride (*r* = 0.358 (men) and 0.363 (women)) and HOMA2-IR (*r* = 0.480 (men) and 0.517 (women)); after adjustment for ASF, IPF was still correlated with triglyceride (*r* = 0.353 (men) and 0.348 (women)) and HOMA2-IR (*r* = 0.290 (men) and 0.221 (women)). WC measures TAF reliably, but its association with IPF depends on IPF/ASF ratios that vary by gender and ethnicity.

## 1. Introduction

Abdominal obesity is one component of the metabolic syndrome [1]. Clinically, abdominal obesity is identified by an increase in waist circumference (WC). Increased WC has repeatedly been linked to metabolic risk. It is unclear, however, whether this measure is a correlate of increased risk through its correlation with total abdominal fat (TAF) or a specific, metabolically unhealthy depot of adipose tissue. Many investigators postulate that the key component of body fat underlying the metabolic syndrome is intraperitoneal fat (IPF) or visceral fat [2–7]. Others nonetheless contend that abdominal subcutaneous fat (ASF) is a more important pathogenic factor [8–14]. Since previous studies have shown that IPF and ASF are intercorrelated [15], the more important adipose-tissue compartment underlying the metabolic syndrome is difficult to identify.

The primary aim of this study was to determine the strength of the correlations between WC and TAF, and ASF and IPF measured by magnetic resonance imaging (MRI). These analyses were made for gender in whites, blacks, and Hispanics of the Dallas Heart Study [16]. We additionally correlated SAF and IPF with plasma triglyceride (TG) and homeostatic model assessment of insulin resistance (HOMA2-IR) [17], both accompanying the metabolic syndrome.

## 2. Methods

Details of DHS study recruitment have been published previously [16]. The current cohort consisted of 1538 women (50% black, 29% white, and 21% Hispanic) and 1212 men (50% black, 36% white, and 16% Hispanic) that had measurement of ASF, IPF, and retroperitoneal fat (RPF). DHS study participants of other ethnicities were excluded from the study. All study volunteers gave written informed consent to participate in an Institutional Review-Board-approved study.

Body weight was measured with a portable scale (Ever Weigh, Lithium electronic scale no. 34067, Health O Meter, Bridgeview, IL, USA) to the nearest 0.1 kg. Height was measured with a stadiometer. Subjects were in a standing position with arms on side, legs straight, and knees together, with feet flat pointed outward. Waist circumference was measured at the midpoint between the lower margin of the last palpable rib and the top of the ileal crest using a stretch-resistant tape with a spring providing constant tension. Fasting plasma lipids, glucose, and insulin were measured as described previously [16]. Insulin resistance was estimated with the HOMA2 computer model (HOMA Calculator version 2.2) [17]. Three categories of WC were defined for this study: low, intermediate, and high. Low WC corresponded to <90 cm in men and <80 cm in women; intermediate WCs were 90–101 cm in men and 80–89 cm in women; and high WCs were ≥102 cm in men and ≥90 cm in women. These cut points corresponded to DHS body mass index categories of <25 kg/m^2^, 25–29.9 kg/m^2^, and ≥30 kg/m^2^. Zhu et al. [18] reported essentially the same ranges based on NHANES III data. Measurements of abdominal compartments of body fat were performed using 1.5 Tesla MRI scanners (Intera; Philips Medical Systems, Best, The Netherlands). The entire abdomen from the diaphragm to the pelvis was scanned using contiguous axial 10 mm slices, as previously described [19]. A single MRI slice at the L2-L3 level was used to quantify total abdominal fat (TAF), ASF, IPF, and retroperitoneal fat (RPF) as detailed by Abate et al. [19]. Briefly, the validation of this method to quantify total abdominal fat subregions involved MRI measurements from the 12th thoracic to 1st sacral vertebra calculated from contiguous 10 mm thick slices that covered the entire abdomen. Regression functions were derived that predicted total fat masses in the respective compartments and that correlated best with the single slice measurement at L2-L3 level. Similar analyses were done to validate the measurement in women [15].

## 3. Statistics

Linear descriptive statistics were employed in data analyses. Data are summarized as means ±S.D. or S.E. for metabolic parameters. For data not normally distributed, results are given as medians (with interquartiles), and data were log-transformed prior to parametric statistical comparisons. Comparisons of means of metabolic risk factors among ethnic groups within each gender were done for metabolic parameters using ANOVA with Bonferroni adjustments for multiplicity of testing or in selected cases using a posthoc Fisher *F* test. Pearson's correlation coefficients were determined for analyses of linear associations of waist girth to abdominal fat parameters measured by MRI. Spearman's and partial correlations were also calculated for relating adipose tissue compartments to triglycerides and HOMA2-IR. A SAS version of StatView (version 5.1.26) was employed for the analyses.

## 4. Results

The clinical characteristics of subjects according to ethnicity and gender are shown in Table 1. Mean ages were in the 40's. Mean BMIs ranged from 28.6 to 32.9 km/m^2^ for all groups. Mean WCs ranged from 99 cm to 101 cm for the three groups of men and from 91.4 cm to 100.7 in women; WCs were higher in Black women. In both men and women, Blacks had the lowest TG and Hispanics had the highest. Black men had higher mean HDL-C levels compared to Whites and Hispanics; differences among the three groups of women were less. Black men had lower non-HDL-C levels than Whites and Hispanics, but they had higher systolic blood pressures.

**Figure 1:**
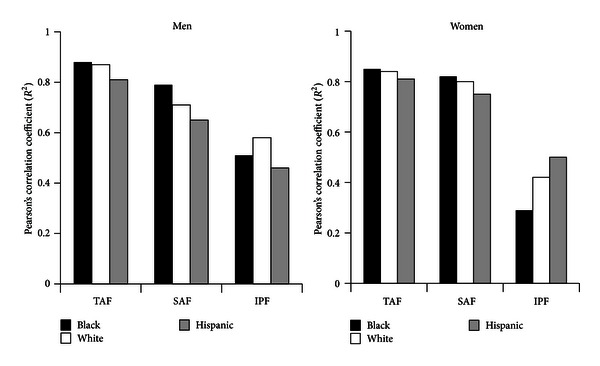
Pearson's correlation coefficients (*r* ^2^) by ethnicity and gender for linear regression analyses of waist circumference versus total abdominal fat (TAF), subcutaneous abdominal fat (SAF), and intraperitoneal fat (IPF). Highest correlations were found for TAF, intermediate for SAF, and lowest for IPF. All correlations were significant at *P* < 0.001.

Pearson's correlations (*R* ^2^) for linear regression analyses between WCs and different abdominal-fat compartments are shown in Figure 1. The highest coefficients of correlation were noted for TAF with progressively lower correlation coefficient with ASF and IPF. IPF was better correlated with WC in men than in women, but still, WC was not a good indicator of IPF. The strength of the correlations was similar within each ethnic group at a *P* < 0.0001.

**Figure 2:**
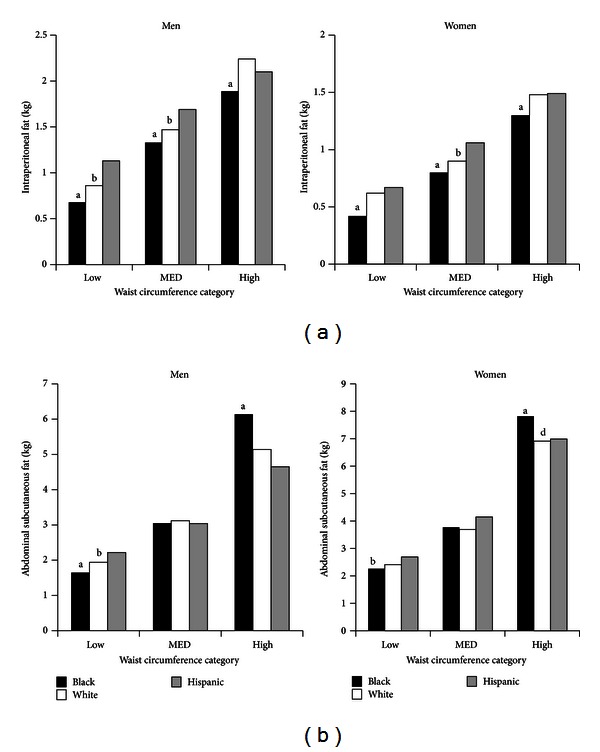
(a) Masses (kg) of intraperitoneal fat (IPF) for each waist circumference category for ethnicity and gender. Amounts of IPF increased for low, intermediate (MED), and high waist circumference categories. ^a^Significantly different from whites and Hispanics (*P* < 0.02); ^b^Significantly different from Hispanics (*P* < 0.02). (b) Masses (kg) of abdominal subcutaneous fat (ASF) for each waist circumference category for ethnicity and gender. Amounts of ASF increased for low, intermediate (MED), and high waist circumference categories. ^a^Significantly different from whites and Hispanics (*P* < 0.02); ^b^significantly different from Hispanics (*P* < 0.02).

Absolute fat masses in IPF and ASF for three categories of WC—low, intermediate, and high—according to ethnicity and gender are presented in Figures 2(a) and 2(b), respectively. Women of all ethnicities had much lower IPF masses than men. Further, in both men and women, blacks had lower IPF masses than whites and Hispanics at all levels of WC (*P* < 0.02); white men with low and intermediate WC had lower IPF than Hispanics (*P* < 0.02); and white women at intermediate WC had lower IPF than Hispanic women (*P* < 0.02). In contrast, men and women had similar patterns of ASF masses for each waist circumference category. Black men had significantly lower ASF than white and Hispanic for those with a low waist circumference, and they also had the highest ASF for those in the highest waist circumference category. The same pattern of ASF fat was noted in black women.

**Figure 3:**
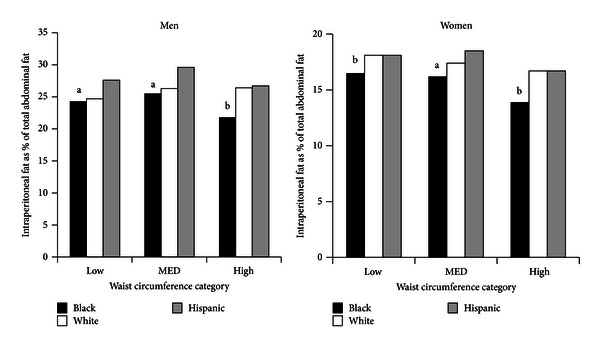
Percentage of intraperitoneal fat (IPF) or total abdominal fat (TAF) for each waist circumference category for ethnicity and gender. Blacks generally had a lower percentage IPF compared to whites and Hispanics. ^a^Significantly different from whites and Hispanics (*P* < 0.02); ^b^significantly different from Hispanics (*P* < 0.02).

Figure 3 displays the IPF as a percentage of TAF. In all ethnicities, higher WCs matched with greater fat masses in each compartment (Figures 2(a) and 2(b)). Although fat masses rose with increasing WCs, the percentage of IPF relative to TAF did not rise for low, intermediate (MED), and high WCs for men or women (Figure 3). In general, blacks had slightly lower percentages of IPF than both other groups.

**Figure 4:**
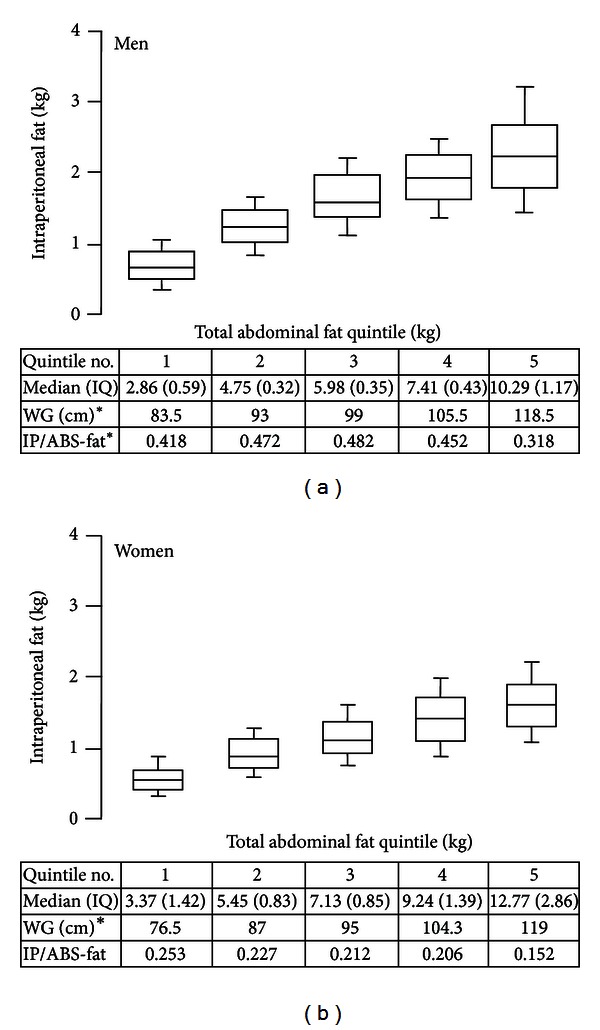
(a) Masses (kg) of intraperitoneal fat (IPF) plotted against masses of quintiles of total abdominal fat for all men. IPF masses increased progressively with TAF, and the distribution of IPF for each category widened. Boxes show mean and one standard deviation; whiskers show 2 standard deviations. The chart gives median values for each quintile, the mean waist girth, and ratio of IPF to abdominal subcutaneous fat. The latter ratio changed a little across ratios except in the highest quintile. (b) Masses (kg) of intraperitoneal fat (IPF) plotted against masses of quintiles of total abdominal fat for all women. IPF masses increased progressively with TAF, and the distribution of IPF for each category widened. Boxes show mean and one standard deviation; whiskers show 2 standard deviations. The chart gives median values for each quintile, the mean waist girth, and ratio of IPF to abdominal subcutaneous fat. The latter ratio changed a little across ratios except in the highest quintile.

In Figures 4(a) and 4(b), ranges of IPF masses are given for quintiles of TAF in men and women, respectively. As TAF rose, so did IPF, showing that most of IPF mass was determined by TAF content. Within each TAF category, nonetheless, there was a range of IPF masses. This range broadened with higher TAF masses, suggesting heterogeneity of IPF response to obesity. In the most obese subjects, IPF masses varied over extremes of about 1.5 kg in men and 1.0 kg in women.

The means and distributions of the IPF/ASF ratio are shown for men and women of the three ethnic groups (Table 2). The distributions were skewed so that mean and 50th percentiles (medians) are not identical. Although mean percentage IPF was relatively constant with increasing WCs, great individual variation was noted across the span of IPF/ASF ratios.

**Figure 5:**
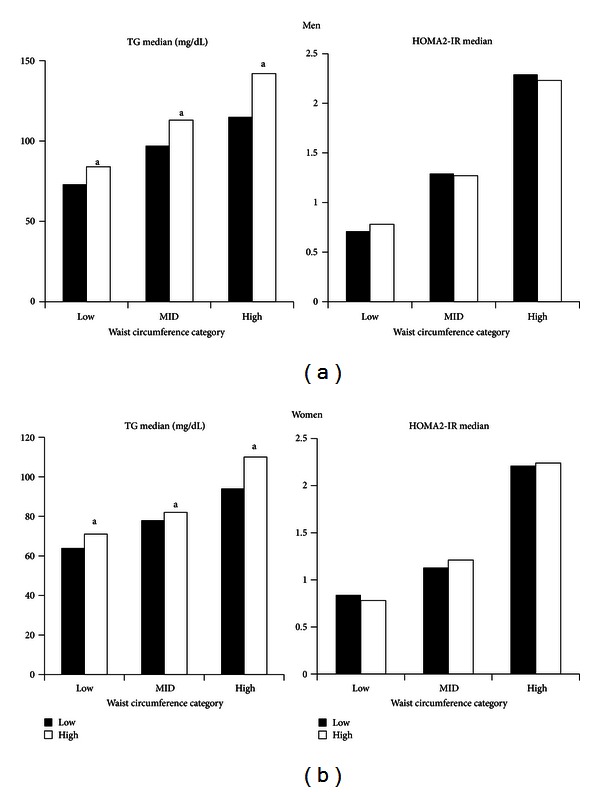
(a) Plasma triglyceride (TG) and HOMA2-IR for upper and lower halves of the intraperitoneal fat/abdominal subcutaneous fat ratios for all men. For TG, those with the high ratios had significantly higher triglyceride in each waist circumference category (^a^ *P* < 0.05). For HOMA2-IR, there were no differences between higher and lower ratios. (b) Plasma triglyceride (TG) and HOMA2-IR for upper and lower halves of the intraperitoneal fat/abdominal subcutaneous fat ratios for all women. For TG, those with the high ratios had significantly higher triglyceride in each waist circumference category (^a^ *P* < 0.05). For HOMA2-IR, there were no differences between higher and lower ratios.

To examine whether differences in IPF/ASF ratios affect plasma TG or HOMA2-IR, ratios were split into upper and lower halves and were related to these metabolic measures. The results for men are given in Figure 5(a). On the whole, for the three WC categories, greater IPF/ASF ratios associated with higher TG levels. In contrast, differences in ratios had little or no influence on HOMA2-IR. In women, a similar but less pronounced trend was noted for differences in IPF/ASF ratios on TG levels (Figure 5(b)). Differences in ratios again had little or no effect on HOMA2-IR.

**Figure 6:**
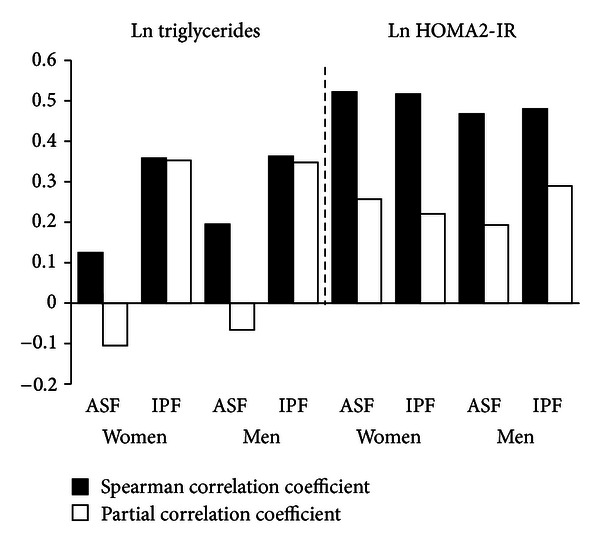
Black bars show Spearman correlation coefficients (*r*) for regression analyses of ln triglyceride and ln HOMA2-IR versus abdominal subcutaneous fat (ASF) and intraperitoneal fat (IPF) in women and men. White bars show partial correlation coefficients after adjustment for the opposite fat compartment. For this analysis, all men and all women were combined. For triglycerides, ASF was modestly correlated but became negatively correlated after adjustment for IPF. IPF was more strongly correlated with triglycerides and remained equally correlated after adjustment for ASF. Both ASF and IPF were strongly correlated with ln HOMA2-IR, but after adjustment for the opposing compartment, partial correlation coefficient was diminished. All correlation coefficients were significant at a level of *P* < 0.001.

To determine whether a more sensitive analysis might identify an effect of IPF on HOMA2-IR, Spearman's correlation coefficients were determined on all men and all women, regardless of ethnicity or WC (Figure 6). In both men and women, ASF was less strongly correlated with TG than was IPF. After cross-adjustment, ASF lost its association with TG levels, whereas IPF did not. In both men and women, ASF and IPF were similarly correlated with HOMA2-IR. After cross-adjustment, the strength of the correlation for each compartment diminished, but IPF remained significantly correlated.

## 5. Discussion

The major findings of this study were the following. First, WC correlated strongly with TAF and ASF, whereas WC less strongly predicted IPF (Figure 1). Second, IPF constituted only about one-fourth of TAF in men and one-fifth in women (Figure 4). Third, for all groups, the distributions of IPF/ASF ratios showed considerable variability among individuals; this explains the relatively low correlations between WC and IPF (Table 2). Fourth, IPF rose progressively with increasing TAF, but at each step of increase, IPF masses varied considerably (Figures 4(a) and 4(b)). Fifth, for all WC categories, persons with higher IPF/ASF ratios had higher plasma TG levels than did those with lower ratios; this relationship was not observed for HOMA2-IR (Figures 5(a) and 5(b)). Even so when all ethnic groups were combined, a positive correlation was uncovered between IPF and HOMA2-IR, which persisted after adjustment for ASF (Figure 6).

IPF, ASF, and the distribution of their ratios were compared to blacks, whites, and Hispanic men and women. Both black men and women had lower median IPF/ASF ratios compared to whites and Hispanics (Table 2). In the three ethnic groups, TAF, ASF, and IPF were similarly correlated with waist girths (Figure 1). In low, intermediate, and high WC categories, both black men and women had lower masses of IPF, compared to whites and Hispanics (Figure 2(a)). In low and intermediate WC categories of men, Hispanics had greater masses of IPF than whites as well as black; at high WG, only black men were different from the other ethnicities. In women, only blacks were consistently different from whites and Hispanics in IFP masses (Figure 2(a)). Black men and women had relatively low ASF mass compared to whites and Hispanics; but in the high WG categories, blacks of both genders had higher ASF mass than whites and Hispanics (Figure 2(b)).

A high WC clearly associates with all metabolic risk factors [1]; and it is commonly believed that WC is a surrogate measurement for visceral adipose tissue [20–22]. The current study revealed that amounts of IPF increased progressively through each category of increasing WC. In this sense, therefore, it can be said that WC is a surrogate for IPF. The current data, nonetheless, indicate that WC is much more strongly correlated with TAF and ASF than with IPF. This being the case, it cannot be assumed that the relation between increased WC and metabolic syndrome is mediated predominantly through a higher IPF.

Abdominal obesity is well recognized to predispose to hypertriglyceridemia [23]. Our study found clear evidence that IPF correlates with plasma TG. For all WC categories and in men and women, those with higher IPF/ASF ratios had higher plasma TG levels. In addition, partial correlation analysis indicated that IPF independently associates with TG levels. The mechanism for this relationship can be readily visualized. Since IPF drains its fatty acids directly into the splanchnic circulation, these fatty acids should add an excess load of lipid on the liver beyond what would be derived from subcutaneous adipose tissue beds. This extra load should translate into higher TG levels.

Several reports suggest that IPF is related to insulin resistance. For instance, Carr et al. [2] reported that intra-abdominal fat is independently associated with insulin resistance, and others found a similar relationship [12, 24, 25]. It might be expected that if IPF causes insulin resistance, a high level of IPF should be a risk factor for type 2 diabetes. Such has been reported [6, 25, 26]. In the current study, IPF appeared to be correlated with HOMA2-IR, albeit weakly. This relationship could not be found when people with high and low IPF/ASF ratios were compared. But partial correlations suggest that higher levels of IPF associate with increased HOMA2-IR independently of ASF. In the light of previous reports, there seems to be little doubt that a positive correlation between IPF and insulin resistance exists.

IPF could be related to either insulin resistance in skeletal muscle or liver. The mechanisms whereby IPF per se could cause skeletal muscle insulin resistance are not readily apparent. An increased release of fatty acids from IPF is one possibility; but amounts must be relatively small compared to the total adipose tissue output of fatty acids. It is thus unlikely that a relatively small increment in release of fatty acids from IPF could substantially worsen insulin resistance in skeletal muscle [27–30].

If IPF increases insulin resistance, it is more likely to be hepatic insulin resistance; this condition is characterized by increased hepatic glucose output. Presumably an increased fatty acid influx into the liver suppresses insulin action, stimulates gluconeogenesis, and raises hepatic glucose output [31, 32]. A report suggests that HOMA2-IR reflects hepatic glucose output more than skeletal muscle insulin resistance [33].

The positive correlation between IPF and HOMA2-IR thus could be mediated through fatty acid stimulation of hepatic glucose output. An interesting question is what are the sources of fatty acids reaching the liver? This question has been examined by Nielsen et al. [28]. They found that the contribution of IPF to hepatic fatty acid delivery ranged from <10% to approximately 50% depending on amounts of IPF. The remainder of fatty acid flux to liver derived from subcutaneous adipose tissue. Thus their findings suggest that excess fatty acids from IPF could drive gluconeogenesis and raise HOMA2-IR.

In reference to the association between visceral obesity and metabolic risk factors, it seems important to distinguish between the rise in IPF with total body obesity and the occurrence of excessive amounts of IPF with obesity. The findings of Nielsen et al. [28] suggest that with increasing obesity, a higher percentage of splanchnic flux of fatty acids is derived from IPF. In addition, for any given level of obesity, there is heterogeneity in IPF content; therefore, obese persons with the greatest IPF masses could have the highest splanchnic flux of fatty acids, worsening liver-associated risk factors.

In summary, this study shows that WC correlates with IPF but not strongly. For any given WC, IPF can vary greatly. The two factors affecting this variation are ethnicity and gender. Black men and women have lower median IPF masses than whites and Hispanics. In the high WG category, blacks have both lower IPF and greater ASF, which contributes to the overall variability between WC and IPF. Both IPF and ASF contribute to metabolic risk factors. IPF was found to correlate with both serum triglyceride levels and HOMA2-IR, whereas ASF correlated only with HOMA2-IR. The mechanisms responsible for these latter correlations require further study.

## Figures and Tables

**Table 1:** Subject characteristics.

	Men			Women		
	Black	White	Hispanic	Black	White	Hispanic
Number of subjects	579	434	199	767	449	272
Age (years)	46 (10)^a^	45 (9)^a^	41 (9)^a^	45 (10)	46 (10)	41 (9)^a^
BMI (kg/m^2^)	29.5 (6.8)	28.9 (5.5)	29.4 (4.6)	32.9 (8.3)^a^	28.6 (6.9)^b^	30.9 (7.4)
Waist circumference (cm)	101 (14)	103 (14)^b^	99 (11)	100.7 (17.2)^d^	91.4 (15.8)	94.5 (16.2)
Hip circumference (cm)	106 (14)	106 (11)	102 (8)^a^	115.6 (16.4)^d^	109.4 (14.9)	109.5 (15.3)
Glucose (mg/dL)	108 (52)	98 (29)^c^	104 (34)	103.5 (48.3)	95.4 (31.6)^b^	107.6 (48.5)
Insulin (pmol/L) median (IQ)	85 (103)	75 (82)^b^	91 (83)	106 (94)	68 (75)^b^	103 (100)
HOMA2-IR (%) median (IQ)	1.63 (1.93)	1.42 (1.51)^b^	1.72 (1.58)	3.63 (3.77)	2.23 (2.75)^b^	3.51 (3.60)
Triglycerides (mg/dL) median (IQ)	121 (120)^d^	157 (121)	170 (131)	80 (52)^d^	98 (75)	111 (77)
HDL cholesterol (mg/dL)	50 (15)^d^	42 (10)	42 (10)	54 (15)	55 (17)	49 (12)^a^
Non-HDL cholesterol (mg/dL)	128 (43)^d^	142 (39)	145 (41)	125 (41)	128 (38)	129 (38)
Systolic blood pressure (Hg mm)	132 (18)^d^	127 (13)^e^	124 (13)	120 (15)^d^	124 (16)	129 (19)
Diastolic blood pressure (Hg mm)	79 (10)	79 (9)	76 (9)^a^	80 (10)^d^	76 (9)	78 (9)
% Metabolic syndrome	30.0	33.9	35.0	42.4	31.5	39.3
% Diabetes mellitus	13.2	5.6	9.7	11.3	5.6	10.8

^a^Significantly different from Blacks and Whites; *P* ≤ 0.0002; ^b^significantly different from Blacks and Hispanics; *P* ≤ 0.03; ^c^significantly different from Blacks; *P* = 0.0001; ^d^significantly different from Whites and Hispanic; *P* < 0.0001; ^e^significantly different from Hispanics; *P* < 0.0001.

**Table 2:** Distribution of intraperitoneal/abdominal subcutaneous fat ratio.

	Mean (SD)	10th	25th	50th	75th	90th
All men	0.462 (0.196)	0.247	0.323	0.426	0.568	0.731
Black	0.416 (0.184)^a^	0.222	0.286	0.387	0.505	0.662
White	0.488 (0.195)	0.262	0.353	0.462	0.592	0.759
Hispanic	0.541 (0.200)	0.318	0.405	0.498	0.655	0.837

All women	0.222 (0.091)	0.118	0.157	0.209	0.273	0.334
Black	0.198 (0.085)^a^	0.101	0.138	0.185	0.247	0.309
White	0.248 (0.090)	0.154	0.186	0.234	0.29	0.358
Hispanic	0.247 (0.092)	0.143	0.183	0.226	0.301	0.375

^a^Significantly different from White and Hispanic (*P* < 0.0001).
